# *Diachasmimorpha longicaudata* Parasitism Response to Medfly Host Fruit and Fruit Infestation Age

**DOI:** 10.3390/insects10070211

**Published:** 2019-07-18

**Authors:** Ahlem Harbi, Luis de Pedro, Fernando A. A. Ferrara, José Tormos, Brahim Chermiti, Francisco Beitia, Beatriz Sabater-Munoz

**Affiliations:** 1 Unidad Asociada de Entomología IVIA-CIB CSIC, Centro de Protección Vegetal y Biotecnología, Instituto Valenciano de Investigaciones Agrarias (IVIA), Ctra. Moncada a Naquea km 4.5, 46113 Moncada, Spain; 2 High Agronomic Institute of Chott-Mariem, University of Sousse, 4042 Chott-Mariem, Tunisia; 3 Instituto Federal Fluminense (IFF), Campus Bom Jesus do Itabapoana, 28360-000 Rio de Janeiro, Brazil; 4 Unidad de Zoologia, Facultad de Biologia, Universidad de Salamanca, 37007 Salamanca, Spain; 5 Smurfit Institute of Genetics, Trinity College, University of Dublin, Dublin, Dublin2 D02 VF25, Ireland; 6 Integrative Systems Biology group, Institute for Plant Molecular and Cell Biology (IBMCP) from the Spanish National Research Council (CSIC) and Polytechnic University of Valencia (UPV), 46022 Valencia, Spain

**Keywords:** *Ceratitis capitata*, *Diachasmimorpha longicaudata*, Tephritidae, Braconidae, fruit host cues, host fruit preference, olfactory trials

## Abstract

The parasitoid *Diachasmimorpha longicaudata* (Ashmead) (Hymenoptera: Braconidae) is increasingly being used in integrated pest management (IPM) programs as a biological control agent in order to suppress tephritid fruit flies of economic importance. Innate and acquired behavioral responses—such as pest host fruit preference—of parasitoids can modulate their efficiency in the field and should be taken into consideration prior to parasitoid species’ selection for mass-rearing. We have assessed the influence of medfly-infested (two infestation ages, 1 and 4-d-old) and uninfested fruit species on host preference and efficiency of *D. longicaudata* by using a multistep assay including olfactory, laboratory and semi-field trials. We found that *D. longicaudata* was significantly more attracted to medfly-infested apples for both infestation ages, with the oldest being the most preferred. *D. longicaudata* exhibited a significant preference among the four fruits tested. The implications of these behavioral responses of *D. longicaudata* to medfly host fruits and infestation age are discussed in relationship to its use in IPM programs in the Mediterranean basin area.

## 1. Introduction

The Mediterranean fruit fly or Medfly, *Ceratitis capitata* (Wiedemann) (Diptera: Tephritidae), is a multivoltine tephritid species that is able to feed and reproduce in more than 300 plant species [1]. These ecological characteristics have allowed the expansion of this species in most temperate areas [2], which has caused them to become an international trade concern for fresh fruit export countries. Fresh fruit producing countries have established pest management programs that have been evolving as society has challenged them to provide more sustainable, environmental and human health-friendly methods. Despite this change, many integrated pest management programs (IPM) still rely on the rational use and application of synthetic pesticides. The report of the emergence of medfly resistant populations (i.e., as reported for malathion-resistant populations in the Mediterranean area of Spain in [3]), along with the description of adverse lethal and/or transgenerational adverse effects on non-target arthropods [4,5,6] or even causing the ultimate outbreak of secondary pest species [7], encouraged stakeholders and governmental policymakers to look for a global change towards biological control with invertebrate species (IBCAs (Invertebrate Biological Control Agent) or generally known as BC (Biological Control)). Even under this positive scenario, putative control agents (usually exotic parasitoids) should be studied under the new local environmental conditions to determine their success before inclusion in BC programs [8,9,10]. 

*Diachasmimorpha longicaudata* (Ashmead) (Hymenoptera: Braconidae) is a solitary koinobiont larval-prepupal endoparasitoid of tephritid species [11,12,13,14]. It is considered among the successful parasitoid species currently used in BC programs, despite the controversy of its species status [11,12]. Several mass-rearing facilities are producing closely related (genetically linked) populations that show differences in parasitism performance either by the rearing conditions or by the target tephritid species [15,16]. Females locate third instar larvae of their tephritid hosts inside infested fruits and use their long ovipositors to lay an egg inside them [17]. Host foraging behavior can be affected by plant cues, especially when these cues modulate the fitness of the parasitoid host [17,18,19] or when these plant cues are being modulated by the same phytophagous host [18,20,21,22]. In general, herbivore-induced plant volatiles are used to refine the search for an adequate host patch. However, this may not be the case with experimented female parasitoids which learned how to link specific cues with the presence of suitable hosts. Furthermore, host choice may not obey the hypothesis of preference–performance, as learning and physio-chemical (color, semiochemicals) cues can be included in host localization (reviewed in [23]). Recently, a host fruit hierarchy for *D. longicaudata* linked to a *D. longicaudata* female’s previous oviposition experience under laboratory conditions has been proposed [24]. Whereas in biological control programs *D. longicaudata* adults are usually released at 6–8-days-old individuals with oviposition experience (with host larvae offered within host artificial diet, reviewed in [25]), the natural scenario proves to be a real challenge. In a natural scenario, the availability and abundance of pest host plants, density or dispersion of the host, host-induced odors (fruit secondary chemicals, or microbe-fermentation-derived odors as a result of host larvae feeding inside the fruits), intraguild competition (female competition for a single host patch), release age (specially female age at release day) and abiotic environmental factors (temperature, humidity and light) may also modify female parasitoids’ host choice by influencing the time and space allocated to host foraging. All these factors can have practical consequences when using parasitoids as biocontrol agents to suppress tephritid pests that can be extrapolated to other species. Indeed, released parasitoids can exhibit differential performance on the same polyphagous herbivore depending on the specific pest host fruit being utilized by the herbivore [24]. 

For all these reasons, we postulate that assessing habitat preference in *D. longicaudata* would be more comprehensive if performed as a multistep assay beginning from olfactory behavioral responses, followed by laboratory tests and confirmed in natural-like conditions while considering the factors as mentioned earlier. 

To fill in this gap as well as to provide data of high practical importance for biological control programs, the present work was conducted to assess how medfly larvae availability on fruit, considering four different host fruits, and medfly-developmental time in fruits (induced fruit volatiles) can influence the parasitism percentage, the fertility (number of progeny) and offspring sex ratio of *D. longicaudata*. 

## 2. Materials and Methods 

### 2.1. Insects and Fruits

The medfly colony was maintained on a wheat bran-artificial diet as described in [25,26,27,28]. Briefly, 350 gr of wheat bran-artificial diet (in 20 × 15 × 4 cm trays covered with aluminum foil) was seeded with 0.5 ml of *C. capitata* eggs, and allowed to develop at controlled conditions (each tray was disposed on a rack within a bigger aerated container of 40 × 30 × 40 cm) until pupation. Pupae were collected from the bottom of the container and used to create a new colony-rearing unit. Rearing units consisted of methacrylate boxes (50 × 40 × 30 cm), with two round holes (9 cm diameter) on the top and one (50 × 40) lateral replaced by muslin-covered frames (for egg laying). Adult medflies were introduced as 80 ml of unenclosed pupae and provided with *ad libitum* water and house-hold sugar within the rearing box. Medfly adult diet (4:1, household sugar: yeast protein hydrolysate) was provided every 2–3 days through the top holes [29]. *Diachasmimorpha longicaudata* rearing was initiated with individuals obtained from parasitized pupae of *Anastrepha ludens* (Diptera: Tephritidae), which were provided by the Centro Internacional de Capacitación en Moscas de la Fruta (CICMF), Plantas de Cría y Esterilización de Moscas del Mediterráneo y Mosca Mexicana de la Fruta, Metapa de Domínguez, Chiapas, Mexico, in 2009. Afterward, the *D. longicaudata* rearing was maintained in the Instituto Valenciano de Investigaciones Agrarias (IVIA), Valencia (Spain), using L2–L3 larvae of *C. capitata* from our rearing colony within the larval diet as host [25,26,27,28,30,31]. *Diachasmimorpha longicaudata* and *C. capitata* were reared under constant controlled conditions in environmental chambers (25 ± 2 °C, 65 ± 10% RH and 16:8 (L:D) photoperiod) as described. 

The choice of fruits used in the experiments was based first on their economic relevance in the Mediterranean area, and then on their availability in the market. We used apple (*Malus domestica* Borkh, cv. Royal Gala), orange (*Citrus sinensis* (L.) Osbeck, var. Navel), peach (*Prunus persica* L., var. Nectarin) and clementine mandarins (*Citrus clementina* Ex. Hort. Tan., var. Clemenules), all purchased from local organic suppliers. All fruits were harvested close to the ripening stage and brought to the laboratory within 24–48h after harvest. Fruits were thoroughly washed with chlorinated tap water, allowed to dry for a half hour and stocked in controlled conditions (8 ± 1°C and 50 ± 5% RH) until trial day. When required, fruits were artificially infested with late second instar medfly larvae as described in [32]. Briefly, 10 equidistant holes 5 mm in diameter and 10–15 mm in depth were drilled in each fruit with a puncher, three medfly larvae were placed in each hole (total 30 larvae/fruit), and each hole was closed with its corresponding fruit plug. Artificially infested fruits were stored at 25 ± 2 °C (room temperature) within pest-proof cages (to avoid any putative/accidental infestation by medfly or other pests under assay in the same laboratory) for 24-h to 4-d, depending on the trial. Non-infested fruits were subjected to the same drilling and re-capping procedure, storage (separated from the artificially infested in different rooms) and aging as the corresponding assay, to be used as a control. In all cases, apples were used as the reference fruit for comparisons between treatments, due to their availability throughout the year. 

### 2.2. Olfactory Response Trials

To assess the olfactory response of *D. longicaudata* adults, a series of olfactory assays were performed using a glass Y-tube olfactometer (Analytical Research Systems, ARS lt, Gainesville, FL, USA) of 13.5 cm base with two arms of 5.75 cm in length, and 2.4 cm of diameter, connected to an air pump producing a unidirectional airflow of 150 ml/min from the arms to the base (producing a wind speed of 0.02 km/h). The Y-tube olfactometer was connected to 5-L crystal jars containing the test fruits, and located in a room with controlled conditions (23 ± 2 °C, 60 ± 10% RH, 2,516 lux) (as described in [33]). In the first series, we tested the response of *D. longicaudata* females to two different age-infested apples. We compared 24-h-old infested apples against uninfested ones, 4-d-old infested apples against uninfested, and then 24-h-old versus 4-d-old infested apples. Finally, we compared 24-h-old infested apples, peaches, clementines and oranges in a pair-wise combination, except for the peach vs clementine comparison as per differential ripening stage of both fruits. As indicated above, uninfested fruits were subjected to the same drilling and aging procedures. For all olfactory tests, we used 8-d-old *D. longicaudata* females having previous experience in parasitizing *C. capitata* larvae from the rearing colony. These females were first individually isolated in 10-ml plastic tubes and left for at least two hours in the olfactometer room to adapt to the assay conditions. Each female was tested independently by placing it at the base of the Y-tube using a soft paint brush, and then observing until it had walked at least 3 cm into one of the arms or until 15 min had elapsed [34]. This procedure was repeated until, at least, recording 30 positive responses for each paired combination, flipping the Y-olfactometer arms 180° each 5 tests to minimize any spatial effect on female choice. In addition, after every 10 females, Y-tube, arms, and jars were thoroughly rinsed with soap, water, acetone and air-dried before proceeding with the next batch of 10 females. Negative responses were those in which females did not make a choice within the 15 min period, being classified as ‘non-responder’ and discarded from subsequent analysis.

### 2.3. Laboratory Trials

*Dichasmimorpha longicaudata* host-fruit preference trials were performed in a climatic chamber (in-house built) with controlled conditions of 25 ± 2 °C, 60 ± 10% RH and 16:8 (L:D) photoperiod. Each experimental unit (or batch) consisted of a ventilated clear plastic box (20 × 20 × 40 cm) with three (only one fruit species for no-choice experiments) or six (two different fruit species for dual choice experiments) isolated-infested fruits deposited inside the box. Each infested fruit was isolated in a 200-ml plastic cup containing a thin layer of vermiculite as a pupating substrate. In choice tests, apples were used as reference fruit, testing against clementine, peach and orange. Each trial consisted of five experimental units of each fruit (no-choice) or fruit combination (dual-choice), being replicated three times (with a total of 15 replicates), totaling 45 and 90 fruits for no-choice and dual-choice tests respectively. In each experimental unit, three 6–8-d-old *D. longicaudata* couples (males were 6-d-old, whereas females were 8-d-old) were introduced and allowed to parasitize medfly larvae for seven consecutive days. After this exposure period, from each experimental unit, pupae were counted and isolated in ventilated 150-ml vials, one per each fruit type. Collected pupae were allowed to develop under controlled conditions until the emergence of adult parasitoids and/or medflies (unparasitized pupae). Emerged parasitoids were counted and sexed. Parasitism percentage (total number of emerged parasitoids divided by the total number of recovered pupae; this variable is also known as Emerged parasitoids percentage by [16]), induced mortality (as the percent number of emerged and un-emerged (unecclosed pupae) parasitoids per total number of recovered pupae; note that this variable was referred to by [16] as parasitism percentage), fertility (total number of emerged parasitoids in each batch), and sex ratio (total number of *D. longicaudata* females divided by the total number of emerged parasitoids) were used as suitable variables to measure host preference [16].

### 2.4. Semi-Field Trials

The dual choice assays were performed in a greenhouse to simulate natural conditions offering larger space and distance between parasitoids and hosts as well as a higher number of infested fruits. The experimental unit consisted of a 2 × 1.5 × 4 m insect-proof cabinet containing two transparent top-open plastic boxes (40 × 40 × 40cm) containing different fruit species in each. Water and household sugar were provided *ad libitum* in each cabinet; in addition, raw honey was provided by being spread over 5 × 10 cm filter paper every two days. Each plastic box contained nine medfly-infested fruits deposited on a thin layer of vermiculite, as described above. Three 6–8-d-old *D. longicaudata* couples were released in each experimental unit and allowed to parasitize for seven consecutive days. After this exposure period, open-top plastic boxes within each experimental unit were closed, retrieved to the laboratory, and all pupae were counted and isolated in ventilated 150-ml vials. Collected pupae were allowed to develop under controlled conditions until the emergence of parasitoids and/or medflies. Emerged adults were counted and sexed as indicated for the laboratory trials. Each assay was replicated four times. Parasitism percentage, fertility, and female sex-ratio were determined as indicated by the laboratory trials [16,32].

### 2.5. Data Analysis

For the olfactometer trials, Chi squared goodness of fit test (*χ*^2^) was used to test the hypothesis that the distribution of side-arm choices between pairs of odors affected the female parasitoid olfactory responses in each experiment. Comparisons between treatment groups were performed using a 2 × 2 contingency table (*χ*^2^, df1). For the laboratory and semi-field trials, a 2-sample unpaired t-test (*α* = 0.05) for no-choice assays or a paired t-test (*P* = 0.05) was performed to compare the parasitism percentage, the fertility, and the offspring sex-ratio. The parasitism percentage and female sex-ratio data were transformed to arcsine square root before analysis [16,35,36]. Statistical analyses were performed using Graphpad Prism statistical software version 5.0 (GraphPad Software Inc., La Jolla, USA).

## 3. Results

### 3.1. Olfactory Response Trials

**Figure 1:**
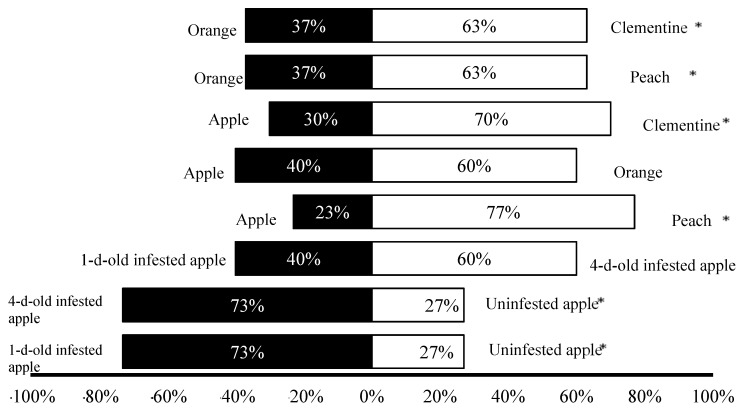
*Diachasmimorpha longicaudata* female response to different odor sources (presented as host fruits), as percentage of responding females. Data were obtained with a Y-tube olfactometer, and different fruit host combinations, including medfly-infested and uninfested (control) fruits, as indicated in the graph. Asterisks represent significant (*P* < 0.05) differences.

In olfactory tests (Figure 1), females of *D. longicaudata* were significantly more attracted to *C. capitata*-infested apple when compared with uninfested ones at both infestation ages (1- and 4-d-old) (*χ*² = 13.07; *P* = 0.0003). A numerically higher number of females preferred 4-d-old infested apples over 1-d-old infested ones, although this was not statistically significant at the α = 0.05 level (*χ*² = 2.40; *P* = 0.12).

When comparing the attractiveness of tested fruit combinations, *D. longicaudata* did not exhibit a significant preference for apple over orange, despite more females responding positively to orange (*χ*² = 2.40; *P* = 0.12). *D. longicaudata* females exhibited significant attractiveness to peach and clementine odors over apple (*χ*² = 17.07; *P* < 0.0001, and *χ*² = 9.60; *P* = 0.0019, respectively). When comparing the choice of *D. longicaudata* between orange, peach and clementine, results revealed a clear preference for peach and clementine compared with orange (*χ*² = 4.26; *P* = 0.03 for both combinations). The *D. longicaudata* female’s preference host fruit hierarchy was peach, clementine >> orange > apple. Despite the lack of statistical significance between orange and apple, we inferred this host fruit preference hierarchy based on the higher percentage of attracted females. 

### 3.2. Laboratory Trials

The assessment of host fruit choice preference of *D. longicaudata* among apple, orange, clementine and peach in laboratory conditions in no-choice and dual-choice tests revealed similar results to those obtained in the olfactory trials. Apple was less attractive than other tested fruits except when challenged against orange (Table 1). *D. longicaudata* females’ response to apple and/or orange did not significantly alter parasitism percentages, fertility, or sex-ratio. When orange and apple were tested together in dual choice tests, the sex-ratio in orange was numerically lower relative to apple, although not statistically significant. Clementine significatively increased the parasitism percentage relative to apple (*P* = 0.040 and *P* = 0.006 for no-choice test and dual-choice test, respectively), also positively affecting fertility and sex-ratio in dual choice tests with apple (*P* = 0.006 and *P* = 0.004 for the fertility and the sex-ratio, respectively). Peach also significatively increased *D. longicaudata* females’ parasitism percentage relative to apple (*P* = 0.0005 and *P* = 0.0047 for the no-choice test and the dual-choice tests, respectively) and fertility (*P* <0.0077 and *P* <0.023 for the no-choice test and dual-choice test, respectively).

In all trials we used apple as a reference to determine the reproducibility of the results, and to allow us to compare *in silico* the fruits that we were not able to compare *in vivo* due to difference in ripening status at the assay time. We did not observe statistical differences in parasitism percentage, fertility or sex-ratio between apple no-choice tests (data not shown), meeting the statistical criteria for comparison among the other fruits. Based on this comparison, we were able to establish a *D. longicaudata* female’s preference and performance in host fruit hierarchy as peach, clementine >> orange and apple, as for the olfactometer assay. 

### 3.3. Semi-Field Trials

In this semi-field assay, all studied parameters revealed a significant preference of D. longicaudata for infested orange, clementine, and peach over apple (Table 2). The parasitism percentage and fertility were significantly higher on orange, clementine and peach than on apple (P = 0.0031, P = 0.0020 and P = 0.0016 for parasitism percentage, and P = 0.0040, P = 0.0008 and P = 0.0011 for fertility, respectively). The sex-ratio (offspring females) responded variably in our reference fruit (apple), with significant opposite results (Table 2; orange P = 0.0111; clementine P = 0.0098). These results could be due to the low percent parasitism and fertility observed for this fruit in the semi-field conditions. 

Based on this comparison, in semi-field trials we established D. longicaudata female’s preference and performance in host fruit hierarchy as orange, clementine, and peach >> apple. 

## 4. Discussion

Apples, clementines, oranges, and peaches constitute some of the preferred hosts for medfly females, becoming economically relevant in the Mediterranean region, due to its quarantine status for many importing countries. With an increasing list of active ingredients banned by the European Union, the control of this economically important tephritid species relies on the application of IPM measures based on phytochemicals, area-wide application of sterile insect technique, and on the importation and release of biological control invertebrate agents. Spain and Tunisia established a collaborative project to import and release the braconid parasitoid *D. longicaudata,* following the European policy [9]. Although this braconid species has long been studied [10,11,13,16,17,36], some points of its biology deserve further research. One of these points involves its parasitism behavior (linked to environmental and learning cues) that would affect its establishment success when reared in one species host and released in a new environment to control a second host species. Thus, our introductory project required some preliminary studies to determine the putative non-target effects, its ability to overwinter or to survive under extreme climatic conditions [32], and most importantly, to determine its success as a biological control agent against the medfly under Mediterranean climatic conditions as well as the most appropriate mass-rearing and release conditions [10,37]. In this study, we determined by multistep assays the effect of four medfly fruit hosts (apple, clementine, orange, and peach), and/or the infestation age, on the medfly parasitism efficacy of *D. longicaudata* in the Mediterranean area. 

With olfactory trials, we demonstrate that *D. longicaudata* was able to distinguish medfly-infested from uninfested and mechanically damaged fruits. Although not statistically significant, numerically *D. longicaudata* females responded preferentially to longstanding infested fruits (4-d-old; at 60%) over recently infested ones (1-d old; at 40%). Considering the absence of visual and physical contact between tested fruits and female parasitoids, it was deduced that *D. longicaudata* females used the medfly larval feeding-induced chemicals for host patch localization and exploitation, as previously noted [17,38]. Other volatile compounds released by the uninfested ripe-rotten oranges or guava fruits such as acetaldehyde, ethanol or acetic acid were also attractive to *D. longicaudata* females [17,39,40,41,42]. As infested fruits become rotten more rapidly than uninfested ones due to the feeding activity of larvae, the release of volatiles will increase with the age of the fruit; to distinguish between them, we used uninfested but damaged fruits. Even with this mechanical damage, only the medfly-infested fruits were significantly attractive to *D. longicaudata* females, which is consistent with the use of medfly larvae-induced volatiles for patch localization, which occurs when providing used artificial medium (a medium that hosted host larvae) [22,42]. In previous work, para-ethylacetophenone was identified as the compound released by the larvae of several tephritid species, that is responsible for enhancing host searching behavior in *D. longicaudata* females [22]. Despite having an identified compound used for host location, it is challenging to determine an easy application system to artificially enhance host foraging in pre-release females. In several programs, the pre-release treatment of *D. longicaudata* only includes oviposition experience with hosts within artificial rearing diet. However, based on results obtained here (fruit preference sequence and infestation stage), we would suggest replacing the pre-release oviposition experience from an artificial rearing diet to a sequence of host-infested fruit-species targeted within the application area to increase *D. longicaudata* female’s fertility and fecundity, at the same time avoiding the observed superparasitism [13,16,35,43]. Tephritid-induced volatiles not only affect the foraging behavior of parasitoids, it seems that they are also involved in mating [21] and in dispersion ability [44,45,46], reinforcing the idea of pre-release treatments for enhancing parasitoid success [47]. 

The use of a reference fruit, along with the olfactory comparison, allowed us to establish a host-fruit attraction hierarchy (peach >> clementine, orange >> apple) comparable with other studies and with other parameters, which rendered a nearly similar parasitism percentage and fertility hierarchy ((clementine>orange), peach> apple) in laboratory and semi-field trials. A host fruit preference hierarchy was also reported between fig and apple for *D. longicaudata* [24]. However, other authors state that there is no association between preferred host fruit and parasitism percentages, although the host-fruit hierarchy is related to fruit fly preference in a density-dependent manner, and linked to the nutritional value of the fruit host [16,18,48,49,50,51,52,53]. The host fruit hierarchy here established (peach, clementine, orange >> apple) nearly resembles the natural ripening status of these commodities in the study area, being a positive point for the improvement of pre-release treatments, as mentioned earlier. 

With regard to the citrus species tested here (clementines and oranges), the differential number of oil sacs in flavedo, albedo thickness, juice vesicles or segment wall thickness, seems to affect tephritid egg-to-larva survival and larval movement within the fruit, also affecting the volatiles cues produced [49,50]. Similar to other parasitoid species, *D. longicaudata* also uses visual (color) and physical signals, especially vibratory signals produced by the host larvae, to locate them within the host fruit [52,53,54]. If the fruit texture alters these vibratory signals, females should spend more time to first locate the host fruits and then to “hear and sense” the larva inside them. As explained previously, the physical and chemical barriers associated with orange and clementine fruits could alter the vibratory signals and odor cues produced by the tephritid larva). Alteration of vibratory signals could, in fact, affect the parasitism percentage, fertility and offspring sex ratio, explaining the differences observed between laboratory and semi-field trials. One major conclusion that can be drawn from this part of the work is that modifying the pre-release learning experience of *D. longicaudata* females (providing them with the target fruit species to be protected) will enhance their foraging success, increase BC success and help in their establishment in the new area [9,13,55,56]. These trained females would also respond to the same host within different fruit cues, increasing the success of BC. The obtained knowledge of this behavioral response along with the functional response already determined, will aid in the development of *augmentoriums* in IPM sensitive areas (areas of ecological value in which chemical treatments are forbidden, that could act as parasitoid reservoirs with multiple host-fruit species) [9,13,55,56]. These *augmentoriums* should be protected and further studied, as they could become the foci of small parasitoid releases in order to serve as a reservoir for increasing parasitoid diversity or as an overwintering shelter for these exotic species.

## 5. Conclusions

In conclusion, our results provide data of practical importance for *D. longicaudata* adaptation to mass-rearing and release in the Mediterranean area by providing information about how host-fruit cues affect host foraging behavior in line with the international required pre-importation studies [9,13,57]. The outstanding host search and foraging ability shown by *D. longicaudata* within the host-fruit hierarchy, which matches the fruit ripening sequence in the Mediterranean area, make this species the perfect candidate for biological control programs in the area, while simultaneously enhancing its presence in the *augmentoriums*. The development of these augmentoriums would also benefit its long-term service as a protective agent against some of the Tephritidae invasive species that are menacing the borders, such as *Bactrocera zonata* or *B. cucurbitae*. However, further research is required to determine their ability to overcome the arid season and their dispersion ability, for future inclusion in the BC program of tephritid fruit flies in the Mediterranean area. 

## Figures and Tables

**Table 1:** *Diachasmimorpha longicaudata* mean medfly parasitism percentage (mean ± S.E.), fertility (mean ± S.E.) and sex ratio (mean [females/(males+females)] ± S.E.) comparing different fruit species in no-choice and dual-choice test under laboratory conditions. Asterisks indicate significant differences.

		No-choice test			Dual choice test		
		Parasitism (%)	Fertility	Sex-ratio	Parasitism (%)	Fertility	Sex-ratio
Apple/Orange	Apple	12.92 ± 2.61	8.87 ± 1.63	33.42 ± 6.95	11.47 ± 2.19	8.60 ± 1.70	21.14 ± 4.80
	Orange	12.10 ± 2.46	7.93 ± 1.66	33.02 ± 8.83	11.72 ± 1.93	7.20 ± 1.17	35.92 ± 6.44
	*t*	0.23	0.4	0.08	0.09	0.68	1.82
	df	1, 28	1, 28	1, 28	1, 28	1, 28	1, 28
	*P*	0.821	0.691	0.937	0.932	0.503	0.081
Apple/Clementine	Apple	12.47 ± 2.83	9.53± 2.20	23.50 ± 7.97	4.86 ± 1.22	3.73 ± 0.95	5.77 ± 5.41
	Clementine	21.87 ± 3.33	16.20± 2.63	29.57 ± 5.56	18.14 ± 4.06	12.60 ±2.70	32.46 ± 5.63
	*t*	2.15	1.94	0.63	3.13	3.10	3.33
	df	1, 28	1, 28	1, 27	1, 28	1, 28	1, 28
	*P*	0.040*	0.061	0.533	0.006**	0.006**	0.004**
Apple/Peach	Apple	8.85 ± 1.79	6.20 ± 1.38	52.85± 8.13	5.98 ± 1.23	3.73 ± 0.81	36.06 ± 9.85
	Peach	19.27 ± 1.94	11.13 ± 1.02	48.55 ± 6.79	14.19 ± 2.30	7.13 ± 1.12	46.00 ± 6.53
	*t*	−3.95	−2.87	0.41	−3.15	−2.46	-0.85
	df	1, 28	1, 28	1, 28	1, 28	1, 28	1, 28
	*P*	0.001**	0.008**	0.688	0.005**	0.023*	0.402

**Table 2:** *Diachasmimorpha longicaudata* mean medfly parasitism percentage (mean ± S.E.), fertility (mean ± S.E.) and sex ratio (mean [females/(males+females)] ± S.E.) in a dual choice test under semi-field conditions. Asterisks indicate significant differences.

		Dual-Choice Test		
		Parasitism (%)	Fertility	Sex-ratio (%)
**Apple/Orange**	Apple	2.62 ± 0.72	5.92 ± 1.63	80.22 ± 6.77
	Orange	15.34 ± 3.37	35.00 ± 8.03	51.79 ± 7.24
	*t*	−3.69	−3.55	2.85
	df	1, 22	1, 22	1, 17
	*P*	0.003**	0.004**	0.011*
**Apple/Clementine**	Apple	0.81 ± 0.55	1.83 ± 1.24	6.33 ± 4.84
	Clementine	6.55 ± 1.42	13.25 ± 2.50	32.75 ± 5.62
	*t*	3.78	4.09	3.02
	df	1, 22	1, 22	1, 13
	*P*	0.002**	0.001**	0.010*
**Apple/Peach**	Apple	2.79 ± 0.92	6.75 ± 2.33	29.64 ± 10.99
	Peach	14.58 ± 2.83	32.08 ± 5.86	36.43 ± 5.05
	*t*	−3.97	−4.02	−0.56
	df	1, 22	1, 22	1, 21
	*P*	0.002**	0.001**	0.584
